# Utility of Handheld Ultrasound in Identifying Millimeter-Sized Vasculature in Living and Cadaveric Subjects

**DOI:** 10.7759/cureus.67383

**Published:** 2024-08-21

**Authors:** Drew A Thibault, Connor Ellis, Josh S Lencke, Karen M Frieswyk, Laurieanne D Hemric

**Affiliations:** 1 Department of Anatomical Sciences, Liberty University College of Osteopathic Medicine, Lynchburg, USA

**Keywords:** carpal tunnel syndrome, cadaveric dissection, variant anatomy, ultrasound imaging, cadaveric imaging, carpal tunnel, persistent median artery, handheld ultrasound

## Abstract

Objective

This study aims to investigate the utility of handheld, Bluetooth-capable ultrasound in identifying millimeter-sized vasculature in both living and cadaveric subjects.

Methods

Using handheld, linear ultrasound probes, the carpal tunnel of 87 living individuals (174 forearms) was assessed for the presence of a persistent median artery (PMA). Radial, ulnar, and persistent median arterial diameters were measured, along with forearm circumference. Using the same probes, 46 cadaveric forearms were assessed for the presence of a “potential” PMA. Those same forearms were subsequently dissected to confirm the presence of the artery.

Results

A PMA was identified in 3.4% of individuals (1.7% of forearms). Radial, ulnar, and persistent median arterial diameters were 2.12 ± 0.40 mm, 1.89 ± 0.41 mm, and 0.82 ± 0.33 mm, respectively. The radial artery was significantly larger than the ulnar artery (p < 0.0001). In cadaveric subjects, four “potential” PMAs were identified by pre-dissection ultrasound. Upon dissection, only one of the “potential” PMAs was confirmed, and three previously unidentified PMAs were identified.

Conclusions

The prevalence of PMA in living subjects was lower than previously reported. Additionally, handheld ultrasound had low accuracy in identifying PMAs in cadavers prior to dissection. This could be an indication that current handheld ultrasound lacks the sensitivity to identify millimeter-sized vasculature, such as a PMA. In both populations of subjects, key, non-anomalous anatomy was readily seen, indicating the utility of handheld ultrasound in the proper context.

## Introduction

Ultrasound technology has advanced rapidly since its introduction in the mid-1900s [1]. While the ability of handheld ultrasound with Bluetooth connection to clearly identify normal anatomy, especially musculoskeletal anatomy, is well-established, few studies have investigated the utility of handheld ultrasound in imaging anomalous, potentially sub-millimeter vasculature in living or cadaveric subjects [2]. To investigate this literature gap, we evaluated the carpal tunnel of living and cadaveric subjects for the presence of a forearm embryological remnant, the persistent median artery (PMA). Although commonly terminating in the antebrachium, the PMA can be present in a palmar pattern (palmar PMA), which travels with the median nerve through the carpal tunnel and supplies blood to the palm [3,4]. Clinically relevant due to its association with carpal tunnel syndrome [5,6], a palmar PMA can also be the sole blood supply to palmar digits [3]. The inability to accurately diagnose its presence preoperatively could cause damage, leading to necrosis and the need for amputation. This study aims to report the prevalence of a PMA detected by handheld ultrasound in a non-clinical sample of living subjects and then compare it to previous prevalence studies in an effort to evaluate the sensitivity of handheld ultrasonography for identifying small, anatomically variant vascular anatomy in living subjects and investigate the utility of handheld ultrasonography in identifying millimeter-sized, anatomically-variant vasculature (i.e., palmar PMA) in cadaveric subjects. This article was previously presented as a meeting abstract at the 2024 Liberty University College of Osteopathic Medicine Research Day on January 12, 2024, and at the 2024 Virginia College of Osteopathic Medicine Research Day on February 16, 2024.

## Materials and methods

Living subjects

For this study, 87 adults from a non-clinical setting voluntarily participated in ultrasonographic imaging of the distal forearm and proximal palm. Both upper limbs (n = 174) were used in identification and measurement of the palmar PMA, radial artery, and ulnar artery. Subjects ranged from 18-62 (median = 25) years of age and were almost equally divided between male and female (n = 44 and n = 43, respectively).

**Figure 1:**
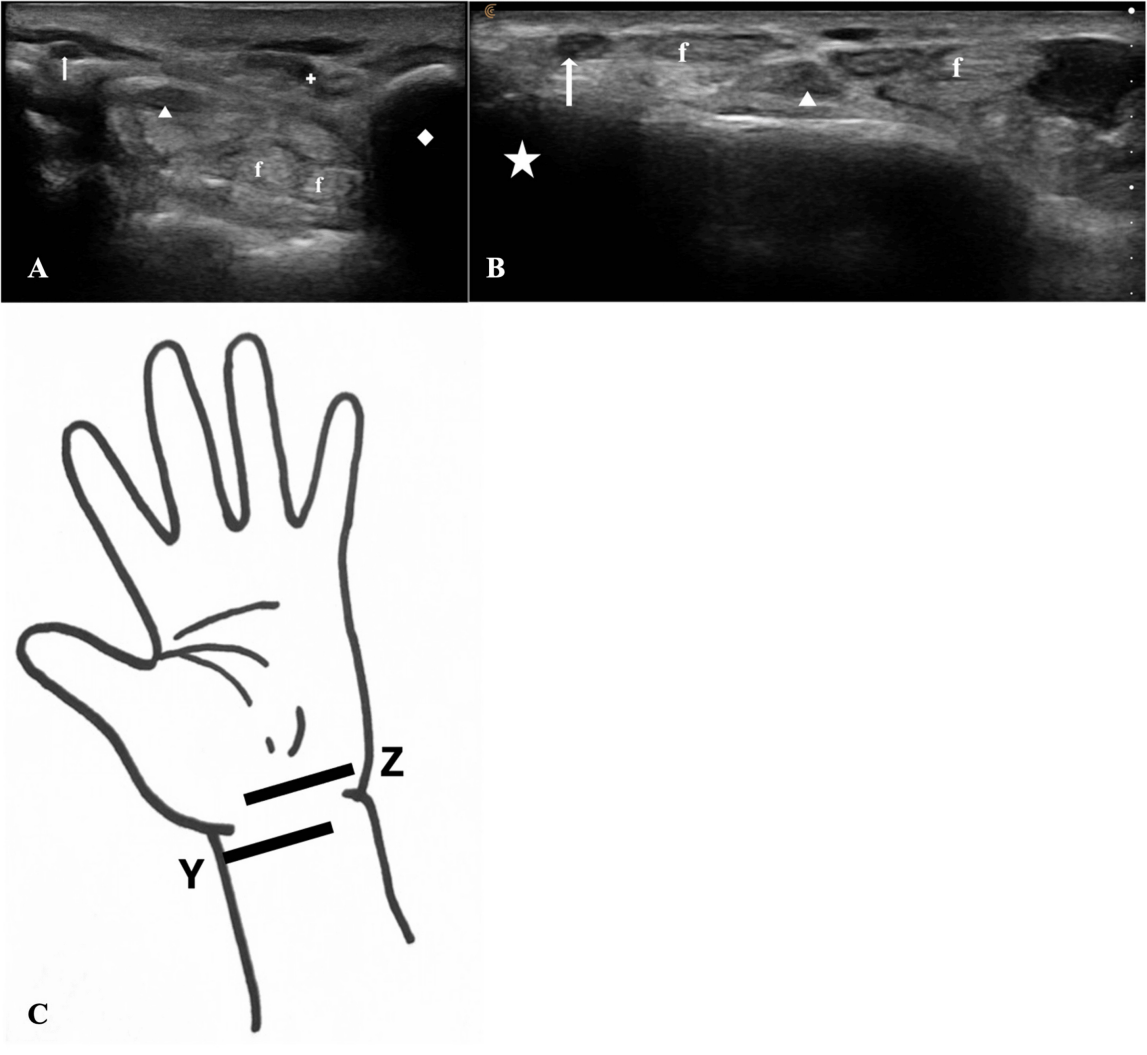
Ultrasonographic view of carpal tunnel in a living subject Ultrasonographic view of the carpal tunnel at the level of the radial styloid process (A) and at the level of the pisiform bone (B) in the transverse plane. C: Drawing of hand indicating relative transducer position for ultrasound imaging of the carpal tunnel at the level of the radial styloid process (‘Y’) and level of the pisiform bone (‘Z’). arrow: radial artery; f: flexor tendon; star: radial styloid process; arrowhead: median nerve; diamond: pisiform bone; crosshairs: ulnar artery

**Figure 2:**
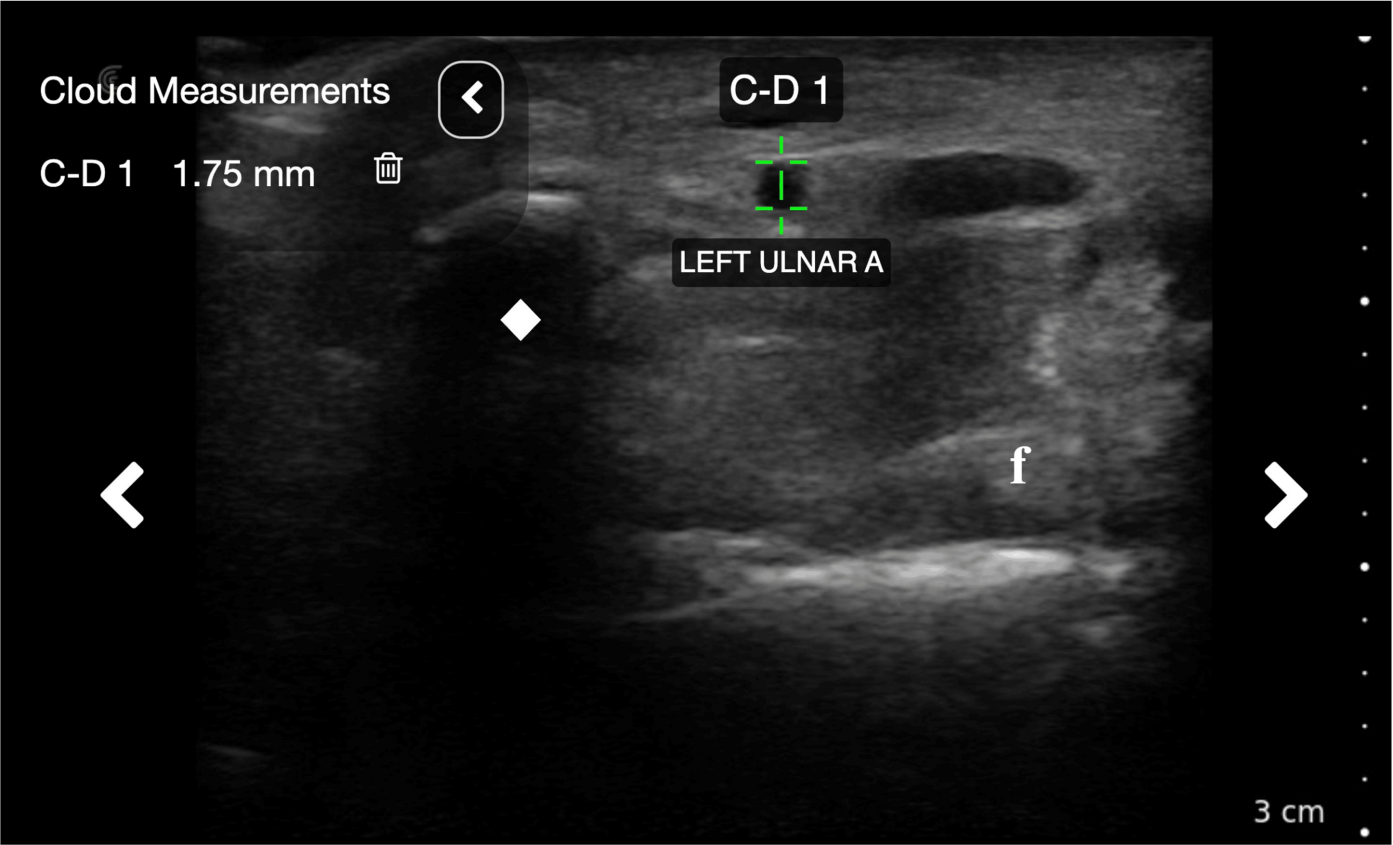
Ultrasonographic arterial measurement Utilization of Clarius virtual caliper function (green crosshairs) to measure internal arterial diameter. The left ulnar artery is demonstrated in this image. diamond: pisiform bone; f: flexor tendon

Ultrasonographic examination was performed using a Clarius ld7 (4-13 MHz) linear ultrasound probe. All ultrasonographic analyses were performed with the linear probe in the transverse plane. The radial artery was identified at the level of the styloid process of the radius (Figure 1), and the ulnar artery was identified at the level of the pisiform bone (Figure 1) [7]. The internal arterial diameter was then measured for each using virtual calipers (Figure 2). The carpal tunnel was then evaluated with color flow Doppler ultrasound at the level of the pisiform bone for the presence of a palmar PMA. The criterion for identification of a palmar PMA was the presence of an anechoic lumen within the carpal tunnel alongside the median nerve. If a palmar PMA was determined to be present, an image was captured, and then internal diameter was measured.

Two investigators were responsible for performing ultrasonographic imaging (C.H.E. and J.S.L.). The presence of a palmar PMA was determined after a review of the images by the research team. Palmar PMA diameter was measured by the lead investigator (D.A.T.).

To explore possible relationships between forearm circumference and forearm arterial diameter, forearm circumference was also recorded bilaterally at the widest part of each participant’s forearm. Measurements were rounded to the nearest 0.5 cm.

The study was conducted at Liberty University College of Osteopathic Medicine, Lynchburg, USA. Prior to examination, study participants gave informed consent for ultrasonographic examination. All procedures were conducted in compliance with Institutional Review Board approval (Liberty University IRB-FY22-23-863).

Statistical analysis for the living subjects was conducted via JMP Pro 17 (JMP, Version 17; SAS Institute Inc., Cary, NC, 1989-2023). Corresponding contralateral arterial diameter data and forearm circumference data were compared bilaterally via two-tailed student’s t-test and simple linear regression. Next, radial and ulnar artery diameters were compared bilaterally, as well as radial and ulnar arteries compared ipsilaterally (within left and right limbs separately), also via two-tailed t-test and linear regression. Lastly, forearm circumference and arterial data were compared between sexes via t-test.

Cadaveric subjects

Using the same ultrasound device, 46 upper limbs (n = 23 cadavers) were evaluated with ultrasonography for the presence of a “potential” palmar PMA. The carpal tunnel was visualized in the transverse plane at the level of the pisiform bone [7]. A “potential” palmar PMA was defined as an anechoic lumen or space alongside the median nerve at the level of the pisiform bone. Ultrasonographic examination was performed by a single researcher (D.A.T.). Images were subsequently saved for future analysis by the research team, who determined whether the anechoic lumen found in each image of a “potential” palmar persistent artery was more likely than not to be an arterial lumen. An expert sonographer was also consulted for confirmation of the research team’s assessments.

In the three months following ultrasonographic imaging of the carpal tunnels, the upper limbs of the same population of cadavers were dissected by students in their first-year gross anatomy medical school course. Following dissection, the presence or absence of a palmar PMA was recorded. A palmar PMA was defined as an artery traveling within the median nerve’s epineurium and into the palm. External arterial diameter of the palmar PMA was also recorded.

## Results

Living subjects

**Figure 3:**
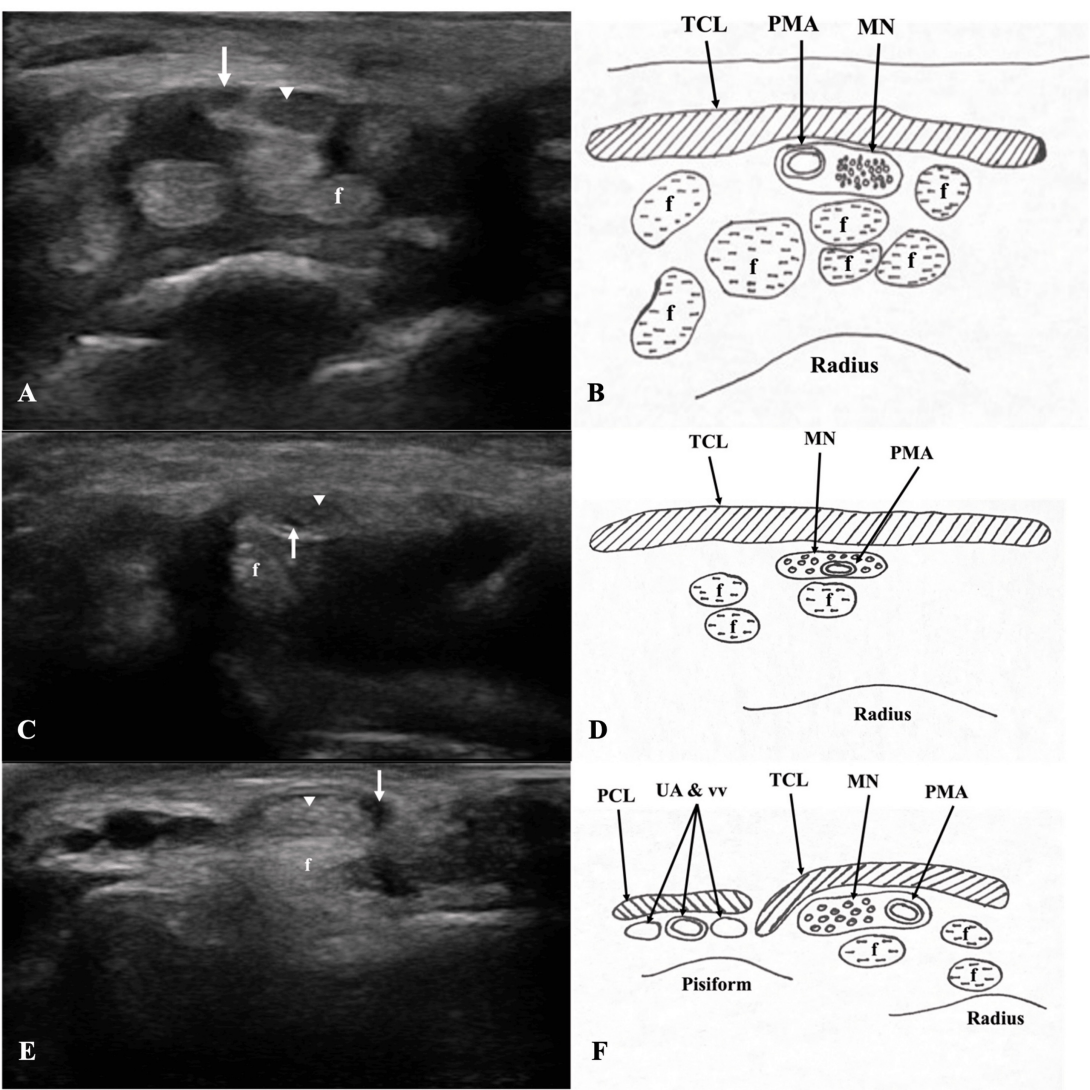
Ultrasonographic view of the persistent median artery in living subjects Transverse ultrasonographic images (A, C, E) and corresponding tracings (B, D, F) of the palmar PMA (white arrows) seen alongside the median nerve (white arrowheads) in the left carpal tunnel of three living subjects. Palmar PMA was found on the ulnar, dorsal, and radial sides of the median nerve in panels A, C, and E, respectively. f: flexor tendons; MN: median nerve; PCL: palmar carpal ligament; PMA: palmar persistent median artery; TCL: transverse carpal ligament; UA and vv: ulnar artery and veins

**Figure 4:**
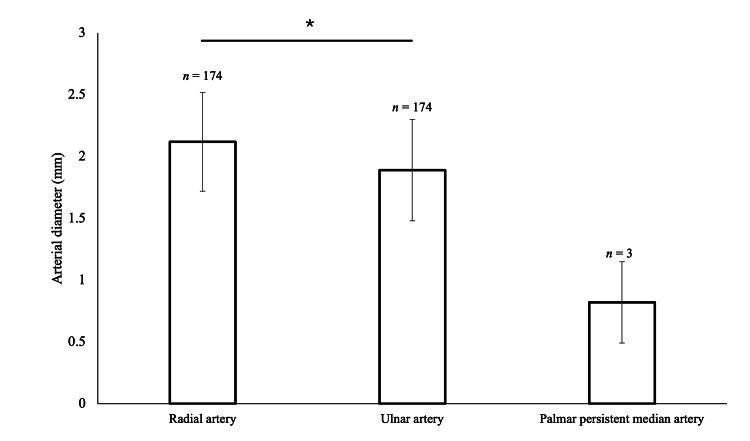
Mean arterial diameters of radial, ulnar, and palmar PMAs +/- 1 SD Mean radial artery diameter (2.12 mm) was larger than mean ulnar artery diameter (1.89 mm) (*, p < 0.0001). Mean palmar PMA diameter was smaller than radial and ulnar arterial diameter; however, statistical significance could not be tested due to the small sample size. PMA: persistent median artery

The prevalence of a palmar PMA detected by ultrasound was only 3.4% (n = 3) in 87 adult living subjects and 1.7% (n = 3) of the 174 upper limbs. All three palmar PMAs were unilateral and were identified within the left carpal tunnel (Figure 3). The mean palmar PMA diameter was 0.82 ± 0.33 mm (range 0.51 - 1.16 mm) (Figure 4). Radial artery diameter was larger (2.12 ± 0.40 mm) than that of the ulnar artery (1.89 ± 0.41 mm) (p < 0.0001, Figure 4 and Appendix 1). Additionally, a significant difference was found when comparing both radial arterial diameter (p < 0.0001) and ulnar arterial diameter between males and females (p < 0.0001) (Figure 4 and Appendix 2). These radial and ulnar diameter data were collected with the expectation that palmar PMA prevalence would be large enough to test our hypothesis about compensatory blood flow; however, palmar PMA prevalence was unexpectedly low, so any comparison could not be made.

Cadaveric subjects

**Figure 5:**
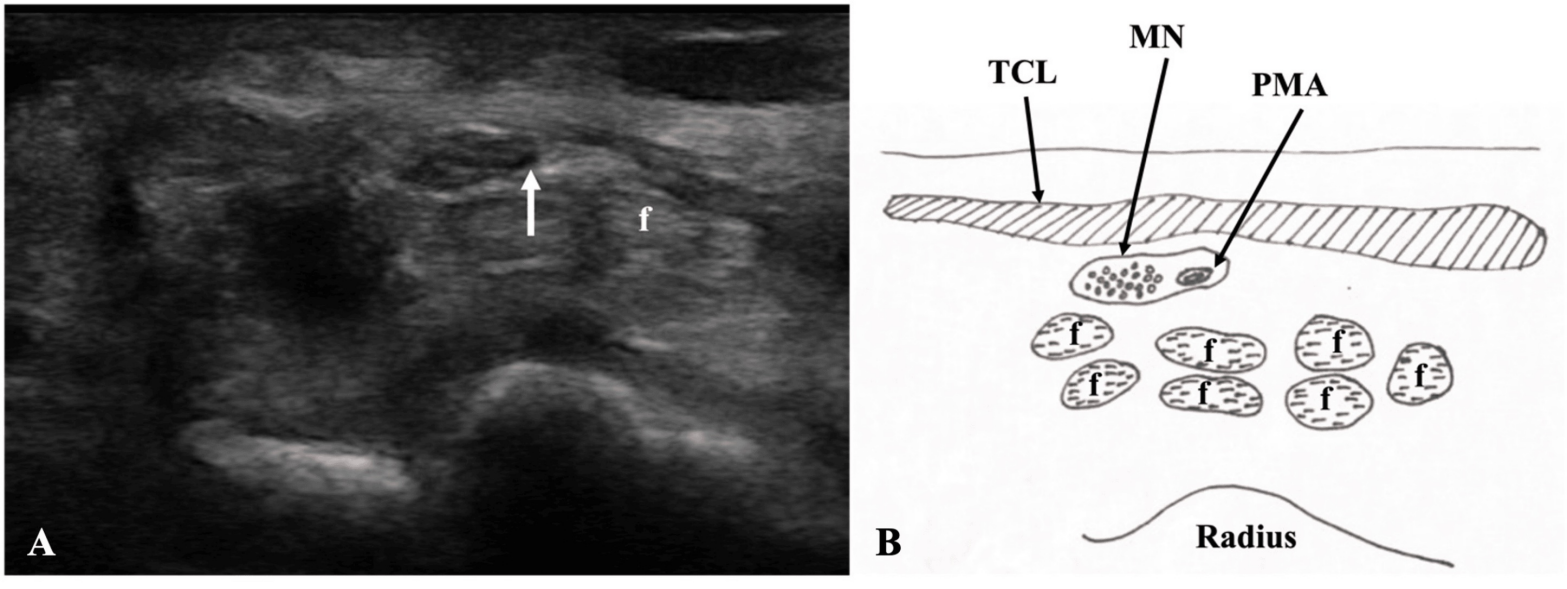
Ultrasonographic view of the persistent median artery in a cadaveric subject Transverse ultrasonographic image (A) and corresponding diagram (B) of the palmar PMA (indicated by anechoic lumen at the tip of the white arrow) seen alongside the median nerve in the left carpal tunnel of a cadaveric subject. Palmar PMA was found on the ulnar side of the median nerve. f: flexor tendons; MN: median nerve; PMA: palmar persistent median artery; TCL: transverse carpal ligament

**Figure 6:**
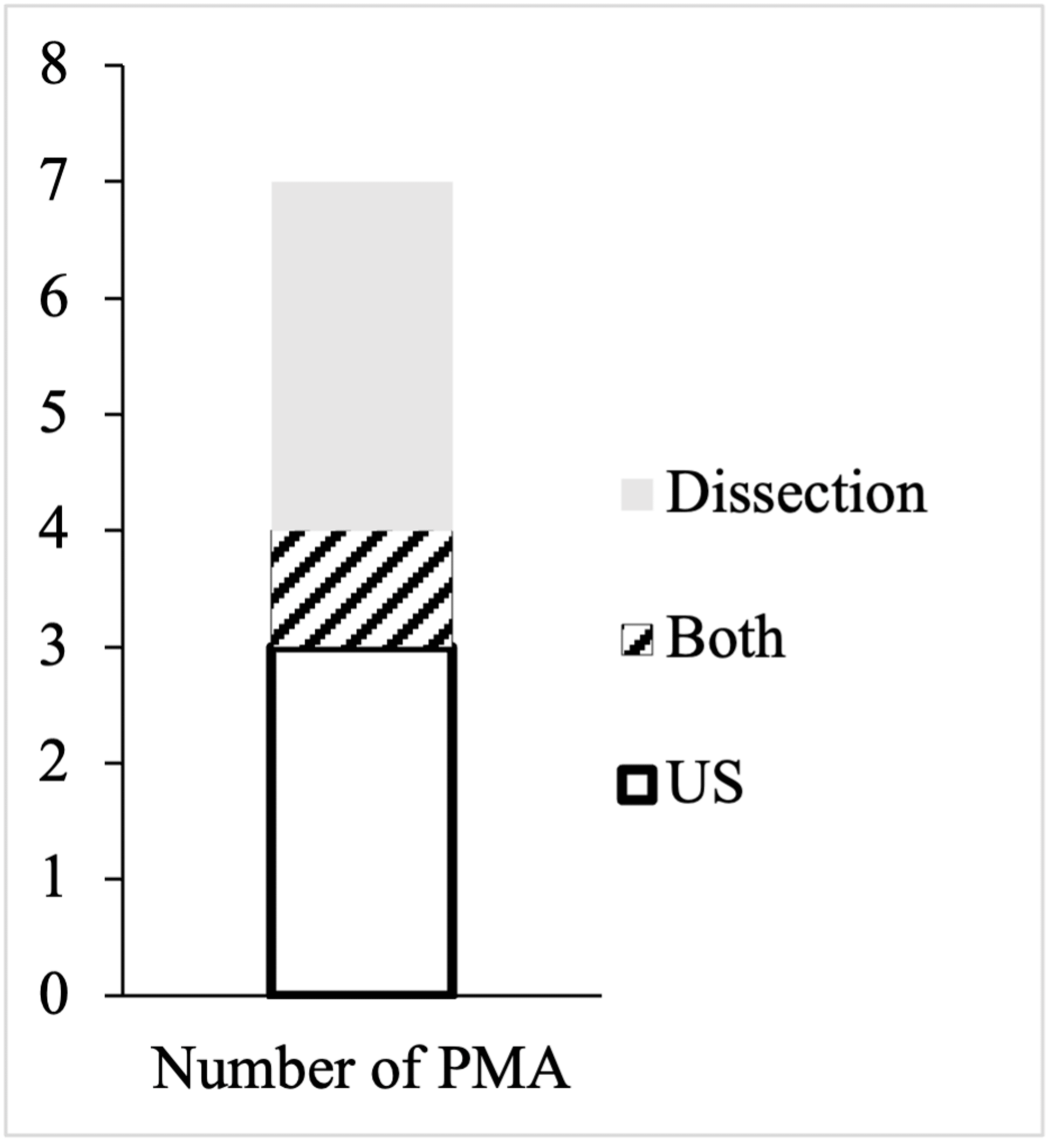
Persistent median arteries identified by ultrasound, dissection, and by both in cadaveric subjects Number of “potential” palmar PMAs identified on ultrasound, number of palmar PMAs identified on dissection, and “potential” palmar persistent median artery confirmed by dissection. PMA: palmar persistent median artery; US: ultrasound

In cadaveric subjects, a “potential” palmar PMA was identified in 8.7% (n = 4) of the upper limbs (Figure 5). Upon follow-up dissection, four palmar PMAs were found in the carpal tunnels of these same 46 upper limbs. However, of these four dissected arteries, only one of them was among the four “potential” palmar PMAs that had been identified by pre-dissection ultrasound. Thus, three of the four palmar PMAs identified during dissection were not identified by pre-dissection ultrasound, and three of the four “potential” palmar PMAs identified by ultrasound were not confirmed by dissection (Figure 6). Mean palmar PMA external diameter was 1.51 ± 0.23 mm (n = 4).

## Discussion

This ultrasound study revealed an unexpectedly lower prevalence of the palmar PMA in living subjects (3.4%) compared to previous studies (8.6%) [6]. By extending our study to cadaveric subjects, we were able to further explore the utility of using handheld ultrasound to detect small-caliber blood vessels such as the palmar PMA. While several studies have reported PMA prevalence via ultrasonography [8-14], to our knowledge, no study has investigated using handheld ultrasound to identify variant carpal tunnel anatomy, such as a PMA. Additionally, this study appears to be the first to utilize ultrasonography to identify variant vasculature prior to cadaveric dissection. We did not aim to quantify the utility of handheld ultrasonography in the identification of small vascular structures; however, the results of our study may allow us to form rudimentary conclusions regarding said utility and provide a framework for future studies.

Living subjects

Our study revealed prevalence rates lower than previously published data. A meta-analysis by Solewski et al. reported an overall cadaveric palmar PMA prevalence rate of 8.6% and a rate of 9.7% (range 3.7 - 20.7%) by ultrasound identification [6]. We propose several reasons for the relatively low prevalence in our PMA data. Our study subjects were randomly selected and drawn from a higher education institution in a non-clinical setting. Study participants were primarily of Caucasian descent, of good health, and were evenly split between male and female (43 and 44 individuals, respectively). Previous studies that reported a PMA prevalence rate using ultrasound had a wide variety of participant populations. Three studies recruited random volunteers [8,11,14], one study had a population of Caucasian males ages 20-30 [13], one study recruited pediatric patients [9], one study had a population of exclusively Chinese individuals [12], and one study recruited individuals of primarily Latin American descent [10]. The three studies with participant selection most closely resembling our study reported prevalence rates of 9% (n = 136) [15], 16% (n = 100) [9], and 19% (n = 100) [11]. Even reasonably large sample sizes appear to yield prevalence rates with noticeable variations.

In addition to population differences, the small size of the palmar PMA and the image quality of handheld ultrasound could make identification of millimeter-sized vasculature difficult to identify. We report the mean internal diameter of the palmar PMA in living subjects as 0.82 mm (n = 3) and the mean external diameter in cadaveric subjects as 1.51 mm (n = 4). Consistent identification of vasculature of millimeter-size may not be possible with the handheld devices used in this study.

We predicted that the presence of a palmar PMA would cause a compensatory change in radial and ulnar arterial diameters. However, the small sample size of PMAs precluded any comparison from being made. The arterial data still yielded interesting results. As expected, right and left forearm circumference were highly predictive of the contralateral forearm circumference (r2 = 0.95); however, right and left radial (r2 = 0.48) and right and left ulnar (r2 = 0.39) arterial diameters were only weakly predictive of contralateral arterial diameter (Table 1). Lastly, we found that radial arterial diameter was significantly larger than ulnar artery diameter. There seems to be no clear relationship between radial and ulnar arterial diameters, as other researchers have shown the ulnar artery to be larger than the radial artery [15,16] and the radial artery to be larger than the ulnar artery [17]. Furthermore, other studies have demonstrated no difference between radial and ulnar diameters [18].

**Table 1:** Comparisons of right and left forearm and arterial measurements

Table 1				
	Right	Left	|t|	r^2^
Forearm circumference (cm)	27.3 ± 3.1	27.1 ± 3.2	p = 0.60	0.95
Radial artery				
Diameter (mm)	2.13 ± 0.42	2.12 ± 0.38	p = 0.78	0.48
Cross-sectional area (mm^2^)	3.71 ± 1.49	3.63 ± 1.31	p = 0.69	0.49
Ulnar artery				
Diameter (mm)	1.87 ± 0.39	1.91 ± 0.42	p = 0.55	0.39
Cross-sectional area (mm^2^)	2.86 ± 1.22	2.99 ± 1.42	p = 0.52	0.38
Mean cumulative arterial cross-sectional area (mm^2^)	6.57 ± 1.99	6.62 ± 2.17	p = 0.88	0.5

Cadaveric subjects

Cadaveric subjects are a highly valuable part of medical education [19]. Using cadaveric subjects, ultrasound is commonly used to allow the practice of nerve blocks and other bedside procedures common to anesthesia [20]. However, little research has been conducted on the efficacy of ultrasonographic imaging in correctly identifying small vascular structures. We predicted that pre-dissection ultrasound imaging would be a sensitive predictor of palmar PMA presence in cadaveric subjects, yet we found a marked difference between our ability to detect “potential” PMAs via pre-dissection ultrasound and the palmar PMAs identified by dissection. This lack of sensitivity could be due to the following reasons: inadequate image quality of cadaveric tissue, absence of blood flow within the vascular lumen, and small size of the average palmar PMA.

Few studies have investigated the quality of ultrasound imaging in living subjects compared to the quality of imaging in cadaveric subjects. Some investigations have sought to compare the images of common injection sites between cadavers and live subjects and have found similar ultrasonographic appearances [21]. Tsui and colleagues compared the use of ultrasound to image airway anatomy in cadavers and living subjects as a means for practicing medical procedures such as laryngeal nerve blocks or cricothyroidotomies, showing similar appearances [22]. While previous studies have shown similar appearance of ultrasound images in living and cadaveric subjects, these studies attempted to identify larger and frequently non-vascular structures. In contrast, Schramek et al. utilized four methods of imaging cadaveric subjects and determined that ultrasound was of the lowest quality [23]. The aforementioned studies provide evidence that ultrasonographic imaging, while of lower quality, is reliable for the identification of large, more commonly identifiable anatomy in cadaveric subjects.

An additional factor impacting image quality is the technique used in tissue preservation. In our study, cadaveric tissue was preserved in a formalin solution, which may not have allowed for the optimal propagation of ultrasonographic waves [24,25], as demonstrated by Sawhney et al. [20]. Additionally, formalin may cause excessive rigidity of cadaveric tissue, creating challenges with correct positioning of the upper limb and further reducing the quality of ultrasound images [24,26].

The sensitivity of pre-dissection ultrasound may also have been compromised by the absence of blood to fill the vascular lumen of cadaveric subjects on ultrasound. Large vessels in cadaveric subjects are typically filled with the formalin solution or with clotted, preserved blood; however, small vessels are often not filled and are collapsed [27]. Identification of a vessel on ultrasound cannot be accomplished without visualization of an anechoic lumen. Additionally, lack of blood flow prevents use of color Doppler ultrasonography to identify vascular structures, making the identification of vasculature more challenging.

The small size of the palmar PMA is another limitation. First, it is possible that a millimeter-sized structure was not readily visible to the naked eye during dissection and was inadvertently removed by student dissection. While students were instructed to dissect with care, this variant artery was not noted in the dissector's instructions. Second, millimeter-sized structures are challenging to identify without magnification and are especially difficult to appreciate in ultrasonography when position and branching patterns are inconsistent. Lastly, if the mean external diameter is typically 1.0-1.5 mm, the internal diameter (the dimension measured by ultrasound) is expected to be even smaller.

The prevalence of the PMA has important clinical relevance. Most commonly, a PMA has been correlated with carpal tunnel syndrome (CTS); however, Solewski et al. showed that the presence of a PMA may, in fact, reduce the risk of developing CTS [6]. While the effects of a PMA on the development of CTS are beyond the scope of this study, the ability to consistently identify PMAs is vital to physicians treating patients with CTS. Failure to do so may result in arterial injury or other complications during treatment of CTS.

While handheld ultrasound is capable of producing high-quality images, handheld devices are generally not as effective as traditional cart-based ultrasound devices [28]. When targeting musculoskeletal structures such as joints and tendons, handheld ultrasound is comparable to cart-based ultrasound when the resolution is the same [29]. Handheld ultrasound devices produce results comparable to cart-based ultrasound in certain clinical scenarios, yet the literature neglects to compare handheld to cart-based ultrasound devices in the identification of the PMA and other anatomical variations. When attempting to visualize small, millimeter-sized vasculatures, handheld ultrasound probes may not provide the same image quality and accuracy.

We acknowledge the following limitations and suggest future directions related to this study. One limitation can be addressed by expanding the sampling population of living subjects to diminish the likelihood of sampling error with an artificially low prevalence rate of palmar PMAs. Additionally, only one form of ultrasound imaging was utilized. Future studies would benefit from comparing handheld ultrasound of the carpal tunnel along with traditional cart-based ultrasound, or MR angiography, of the carpal tunnel to further evaluate the accuracy of handheld ultrasound in identifying millimeter-sized vascular structures. Pre-treatment with a vasodilatory agent such as topical glyceryl trinitrate could be done in future studies to increase forearm vascular diameter prior to evaluation with handheld ultrasonography. Regarding the cadaveric population, limitations include the possibility that PMAs were present on pre-dissection ultrasound imaging and then incorrectly dissected away before those same upper limbs were evaluated by investigators. Another limitation was the utilization of only formalin-preserved cadavers [24,26]. Future studies should be expanded to include pre-dissection ultrasonography of cadaveric subjects who have undergone different preservation techniques, which may further illuminate the accuracy of handheld ultrasound in identifying a PMA.

## Conclusions

While the identification of small, millimeter-sized vascular structures may not be the most optimal use of the form of ultrasonography utilized in this study, the images produced by the Clarius ld7 devices are excellent for the identification of larger, expected anatomical structures (i.e., not anatomical variants). This is true for both living and cadaveric subjects. In living subjects, the images of the carpal tunnel were of high enough resolution to clearly identify the invariable anatomy of the carpal tunnel. Similarly, in cadaveric subjects, larger structures such as the median nerve were identifiable in the carpal tunnel using handheld ultrasound probes. While the form of handheld ultrasonography utilized in this study may not be ideal for the identification of millimeter-sized vascular structures, the quality of the images produced is sufficient for both clinical imaging and medical education in the proper situation.

## Appendices

Appendix 1

**Table 2:** Comparing mean diameter and cross-sectional area of the radial artery and ulnar artery bilaterally

	Radial artery	Ulnar artery	|t|	r^2^
Mean diameter (mm)	2.12 ± 0.40	1.89 ± 0.41	p < 0.0001	0.0308
Mean arterial cross-sectional area (mm^2^)	3.67 ± 1.40	2.93 ± 1.32	p < 0.0001	0.0268
Mean left diameter (mm)	2.12 ± 0.38	1.91 ± 0.42	p = 0.0008	0.0774
Mean left arterial cross-sectional area (mm^2^)	3.63 ± 1.31	2.99 ± 1.42	p = 0.003	0.0669
Mean right diameter (mm)	2.13 ± 0.42	1.87 ± 0.39	p < 0.0001	0.0062
Mean right arterial cross-sectional area (mm^2^)	3.71 ± 1.49	2.86 ± 1.22	p < 0.0001	0.0053

Appendix 2 

**Table 3:** Comparing male and female forearm circumference, radial artery, and ulnar artery diameters

	Male	Female	|t|
Mean forearm circumference (cm)	28.6 ± 6.5	25.8 ± 4.8	p < 0.0001
Mean radial artery diameter (mm)	2.29 ± 0.53	1.96 ± 0.29	p < 0.0001
Mean ulnar artery diameter (mm)	2.07 ± 0.52	1.70 ± 0.32	p < 0.0001
